# Associations between neighbourhood characteristics, physical activity and depressive symptoms: the Northern Finland Birth Cohort 1966 Study

**DOI:** 10.1093/eurpub/ckad215

**Published:** 2023-12-11

**Authors:** Nina Rautio, Marjo Seppänen, Markku Timonen, Soile Puhakka, Mikko Kärmeniemi, Jouko Miettunen, Tiina Lankila, Vahid Farrahi, Maisa Niemelä, Raija Korpelainen

**Affiliations:** Research Unit of Population Health, University of Oulu, Oulu, Finland; Medical Research Center Oulu, Oulu University Hospital and University of Oulu, Oulu, Finland; Research Unit of Population Health, University of Oulu, Oulu, Finland; Department of Sports and Exercise Medicine, Oulu Deaconess Institute Foundation sr, Oulu, Finland; Geography Research Unit, University of Oulu, Oulu, Finland; Research Unit of Population Health, University of Oulu, Oulu, Finland; Unit of Primary Care, Oulu University Hospital, Oulu, Finland; Research Unit of Population Health, University of Oulu, Oulu, Finland; Department of Sports and Exercise Medicine, Oulu Deaconess Institute Foundation sr, Oulu, Finland; Geography Research Unit, University of Oulu, Oulu, Finland; Research Unit of Population Health, University of Oulu, Oulu, Finland; Research Unit of Population Health, University of Oulu, Oulu, Finland; Medical Research Center Oulu, Oulu University Hospital and University of Oulu, Oulu, Finland; Department of Sports and Exercise Medicine, Oulu Deaconess Institute Foundation sr, Oulu, Finland; Geography Research Unit, University of Oulu, Oulu, Finland; Research Unit of Health Sciences and Technology, University of Oulu, Oulu, Finland; Institute for Sport and Sport Science, TU Dortmund University, Dortmund, Germany; Medical Research Center Oulu, Oulu University Hospital and University of Oulu, Oulu, Finland; Department of Sports and Exercise Medicine, Oulu Deaconess Institute Foundation sr, Oulu, Finland; Research Unit of Health Sciences and Technology, University of Oulu, Oulu, Finland; Research Unit of Population Health, University of Oulu, Oulu, Finland; Medical Research Center Oulu, Oulu University Hospital and University of Oulu, Oulu, Finland; Department of Sports and Exercise Medicine, Oulu Deaconess Institute Foundation sr, Oulu, Finland

## Abstract

**Background:**

Due to rapid urbanization, there is a need to better understand the relative roles of residential environment and physical activity in depression. We aimed to investigate whether neighbourhood characteristics are related to the presence of depressive symptoms and whether the association is modified by physical activity.

**Methods:**

This cross-sectional study used the 46-year-old follow-up data (*n *=* *5489) from the Northern Finland Birth Cohort 1966. Data on depressive symptoms, measured by Beck Depression Inventory-II, and self-reported and accelerometer-measured physical activity were included. Neighbourhood characteristics, population density, distance to the closest grocery store, bus stops and cycle/pedestrian paths, distance to the nearest parks and forests, residential greenness and level of urbanicity were calculated using Geographic Information System methods based on participants’ home coordinates.

**Results:**

According to ordinal logistic regression analyses adjusted for physical activity at different intensities and individual covariates, living in a neighbourhood with higher population density and urbanicity level were associated with a higher risk of experiencing more severe depressive symptoms. Higher residential greenness was associated with a lower risk of experiencing more severe depressive symptoms after adjustment for self-reported light and moderate-to-vigorous physical activity, accelerometer-measured moderate-to-vigorous physical activity and individual covariates. Both higher self-reported and accelerometer-measured physical activity were independently associated with a lower risk of more severe depressive symptoms.

**Conclusions:**

Both residential environment and physical activity behaviour play an important role in depressive symptoms; however, further research among populations of different ages is required. Our findings can be utilized when designing interventions for the prevention of depression.

## Introduction

Depression is a commonly occurring disorder, and in severe untreated cases, it can lead to suicide.^1^ The risk of depression differs between sex, age and socio-economic status, and its prevalence varies considerably both within and between countries worldwide.^1^ The aetiology of depression is complex, partly unknown and multi-factorial, including genetic, social and environmental factors.^1^^,^^2^ The role of residential environmental characteristics, including behavioural factors, especially physical activity (PA) and sedentary behaviour, in the development of depression have gained increasing interest in recent decades.^3^^,^^4^

At present, more than 50% of the world’s population lives in urban areas, and the proportion is expected to increase up to 68% by 2050.^5^ The findings concerning the association between urbanization and the incidence of depression have been inconsistent.^6^ Some studies suggested that living in more urbanized areas was associated with an increased risk of depression as compared to living in more rural areas, after adjustment for individual characteristics.^7^^,^^8^ Walters et al.^9^ suggested an association between high population density and prevalence of depression, but more walkable neighbourhoods reportedly reduce the odds of depressive symptoms.^10^ In addition, the presence of healthy food stores, fast food restaurants and health services has been associated with a lower prevalence of depression.^11^ Green space is a fundamental component of neighbourhoods, as higher amount of residential green space has been shown to be associated with lower risk of depressive symptoms.^12^

A higher level of PA is reportedly independently associated with decreased depressive symptoms.^13^ An up-to-date review of observational studies showed that the amount of both customary and moderate-to-vigorous PA (MVPA) was inversely associated with incident depression and the onset of subclinical depressive symptoms among adults regardless of global region, sex, age or follow-up period.^14^ In addition, another recent systematic review indicated that various residential built environment characteristics, such as destination accessibility, land use mix and new infrastructure for active transportation, were associated with increased walking and cycling.^15^ Furthermore, positive associations have been shown between PA, residential green space^16^ and short distance to a variety of destinations.^17^

Overall, exposure to natural environments and higher levels of PA are both independently associated with reduced depressive symptoms. Furthermore, a meta-analysis conducted by Wicks et al.^18^ indicated that engaging in PA in outdoor natural environments is associated with significant reductions in depression compared to PA undertaken in outdoor urban environments. However, studies investigating the potential moderating role of PA in the associations between different neighbourhood characteristics and depression, especially using device-based measurements of PA, are scarce.^19^ In addition, it has been noted that employing both self-reported and device-based methods of PA assessment is needed in research to better understand the associations between PA and health outcomes.^20^

The aim of this study was to investigate whether neighbourhood characteristics are related to depressive symptoms at midlife and whether the possible associations are modified by self-reported or accelerometer-measured PA. We hypothesized that living in an area with good accessibility to services and green or walkable areas are related to lower levels of depressive symptoms. In addition, we believe that the association between neighbourhood characteristics and depressive symptoms varies after including different measures and intensity of PA.

## Methods

### Study population

**Figure 1:**
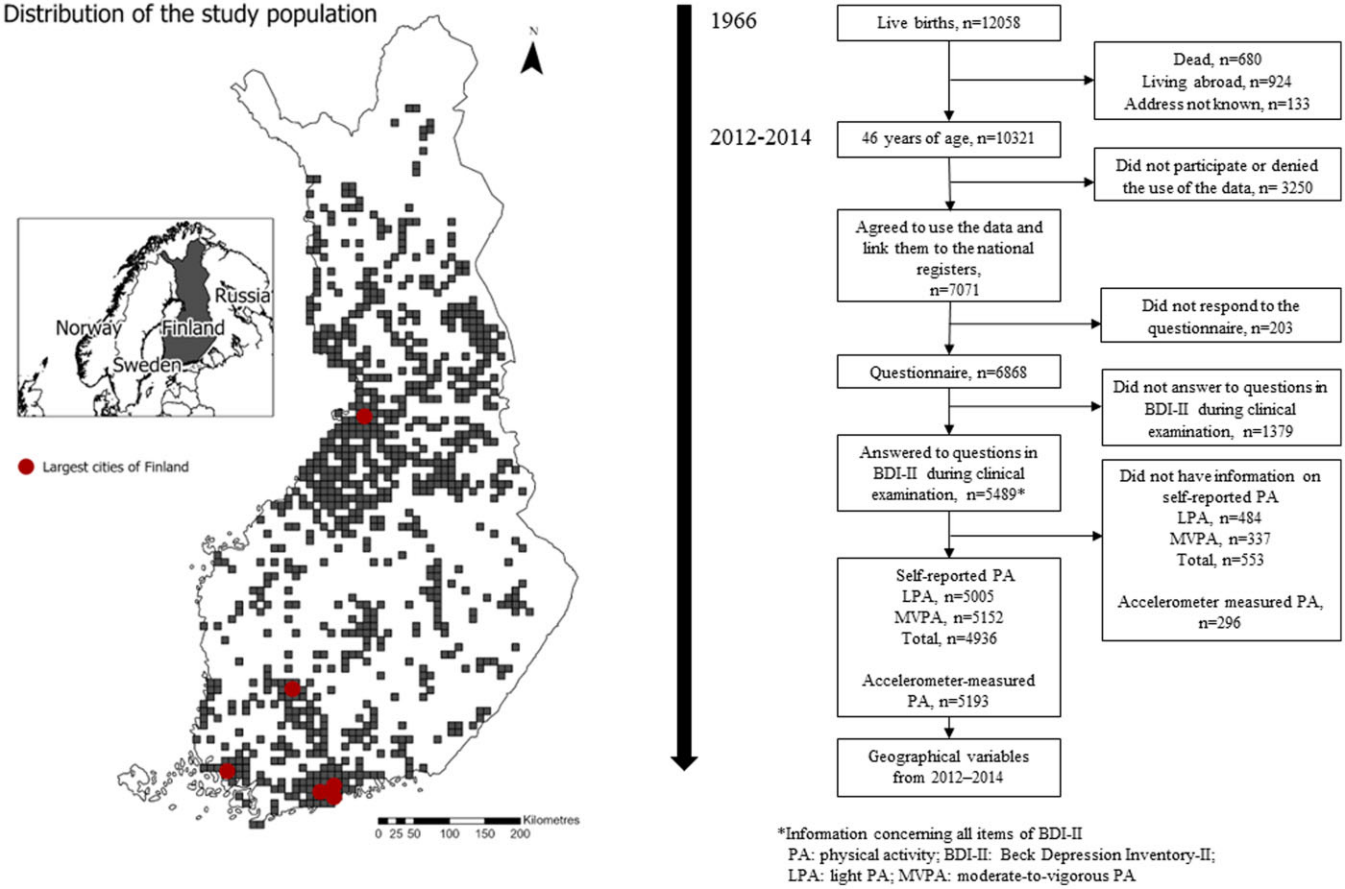
Flow-chart and the distribution of the study population

This study is a part of the population-based Northern Finland Birth Cohort 1966 (NFBC1966) study.^21^ NFBC1966 comprised 12 058 live-born children (96.3% of all births) during 1966 in two former northern provinces of Finland, Oulu and Lapland. This cross-sectional study includes data from the most recent collection at the 46-year follow-up between 2012 and 2014. The data was gathered through questionnaires and clinical examinations. More details on the follow-up, including loss of follow-up and missingness, has been described earlier by Nordström et al.^22^ The study was approved by the Ethical Committee of the Northern Ostrobothnia Hospital District (94/2011), and written informed consent was obtained from all the participants. Of the 10 321 eligible individuals, 7071 (68.5%) approved the use of their data and linkage to the national registers, 6868 (66.5%) filled in the questionnaire during the clinical examination and 5489 (53.2%) provided information on depressive symptoms. All those with information concerning self-reported leisure-time light PA (LPA) (*n *= 5005), MVPA (*n *=* *5152) and accelerometer-measured LPA and MVPA (*n *=* *5193) were included in the study (figure 1). When comparing the final sample to drop-outs regarding sex and register-based marital status and register-based education, we found that 31.4% of those with basic education, half (50.2%) of those with secondary education and 61.1% of those with tertiary education participated in the study. Women (52.4%) and those who were married (52.2%) participated more often than men (42.8%) and those not married (42.9%).

### Depressive symptoms

Information on depressive symptoms was collected during the clinical examination using Beck Depression Inventory-II (BDI-II).^23^ BDI-II is composed of 21 items that are accepted as depressive symptoms, such as pessimism, self-dissatisfaction, loss of appetite, uneasiness, indecision, fatigue, sense of failure, guilt, sleep disorder and social withdrawal. Each answer was scored on a scale value of 0–3. Higher total scores indicated more severe depressive symptoms. Total score of BDI-II was categorized as follows: no depressive symptoms (scores of 0–13), mild depressive symptoms (14–19); moderate depressive symptoms (20–28) and severe depressive symptoms (26–63).^23^ In this study population, the value of Cronbach’s alpha for BDI-II items was 0.92, indicating high internal consistency.

### Neighbourhood characteristics

Neighbourhood characteristics were calculated using ArcMap 10.2 based on the participants’ home coordinates. A circular buffer with a 1 km radius was fixed around each participant’s residency to represent their everyday living environment.^24^ Neighbourhood characteristics were available annually for 2012–2014 and were linked to the cohort questionnaire and clinical data according to participants’ clinical examination date. The information concerning the beginning and ending date of the residence was received from the Digital and Population Data Services Agency. If the accurate starting date of the residence was unavailable but we had the information concerning the month and year, we considered the last day of the previous month as the starting date of the residence.

#### Population density

Population density (inhabitants/0.1 ha) was based on the number of people living in a buffer within a 1 km radius surrounding the cohort members’ homes.^25^ Population data were derived from the Finnish Community Structure Follow up System (FCSFS).

#### Distance to the closest grocery store

The road network from the Finnish National Road and Street Database was used to calculate the distance from the participant’s residence to the focal point (250 m × 250 m), where the closest grocery store (supermarket or store) existed. Data regarding grocery stores were obtained from the FCSFS.

#### Bus stops and cycle/pedestrian paths

Information on bus stops and paths was obtained from the Finnish National Road and Street Database. The number of public transportation bus stops was calculated using a 1 km buffer. The length (m) of cycle/pedestrian paths was measured based on a 1 km buffer.

#### Distance to the nearest parks and forests

Distance (m) to the nearest park and forest as the crow flies was measured using CORINE land cover vector dataset (mapping unit 25 ha) from the Finnish Environment Institute.

#### Residential greenness

Normalized difference vegetation index (NDVI) was used to define the residential greenness in the neighbourhood using a 1 km buffer, which was calculated according to the following equation:

$$\frac{\mathrm{NIR}-\mathrm{VIS}}{\mathrm{NIR}+\mathrm{VIS}}$$

where NIR means the amount of reflected near-infrared radiation and VIS is the amount of visible light. NDVI values vary between −1.0 to +1.0, with higher values indicating denser vegetation. NDVI was based on satellite imagery from the United States Geological Survey. Images with <10% cloud cover were selected, and the months of June to July (2013–2016) were used in the calculation as they represent the greenest months in Finland’s seasonal variation.

#### Level of urbanicity

Urbanicity was characterized by higher levels of population, services and transportation networks in the neighbourhood. It was measured with an index combining standardized z-scores of density of residents, services and intersections within a 1 km buffer.^26^^,^^27^ The number of services was obtained from FCSFS and was based on the sum of amenities for retail (shops, market halls, department stores and commercial centres), recreation (accommodation, restaurants, theatres, cinemas and sports facilities) and office and community (libraries, museums, churches, health care and schools). Street network data were derived from the Finnish National Road and Street Database and included intersections with three or more legs. A higher value indicates a higher level of urbanicity.

### Self-reported PA

PA was self-reported with questions on the frequency (from once a month to daily) and duration (from 0 to >1.5 h a day) of LPA and MVPA during leisure-time.^28^ LPA was defined as causing no sweating or breathlessness and MVPA as causing some sweating and breathlessness. The amount of LPA and MVPA was calculated separately by frequency × duration.

### Accelerometer-measured PA

All study participants attending the clinical examinations were asked to wear a wrist-worn waterproof Polar Active monitor (Polar Electro Oy, Kempele, Finland) for at least 2 weeks (including while sleeping) in the non-dominant hand. Participants were instructed to live their everyday life as usual and not to change their behaviour during the accelerometer data collection. The accelerometer did not provide any feedback to the participants during the data collection. Participants with ≥4 valid days (wear time of at least 600 min/day during waking hours) were included in the analyses.^29^ Polar Active provides estimated metabolic equivalent (MET) values every 30 s based on daily PA.^30^ The accelerometer-measured PA was categorized into five intensity levels based on the cut points provided by the device manufacturer: very light, 1–2 MET; light, 2–3.5 MET; moderate, 3.5–5 MET; vigorous, 5–8 MET; and vigorous+, ≥8 MET.^31^ For all participants, calculations for daily and weekly averages of duration spent in different activity intensity levels (min/day) were conducted. Time (min/week) spent on any activity at an intensity of 2–3.5 METs was categorized as LPA and at an intensity of at least 3.5 METs was categorized as MVPA (min/week).

### Covariates

Sex, marital status, harm avoidance personality trait, education, current smoking and alcohol intake were used as covariates based on earlier literature and preliminary analysis. Marital status was classified as married/cohabiting/registered partnership and single/divorced/widow. Harm avoidance was based on temperament items from Cloninger’s Temperament and Character Inventory.^32^ Education was based on two questions on basic and vocational education and was classified as basic (≤9 years of school and no vocational education or only short course), secondary (vocational school or college degree and/or matriculation examination) or tertiary (polytechnic or university degree). Present smoking was classified as ‘No/Yes’. Alcohol intake was based on questions on frequency of use and typical quantity per occasion of (i) mild drinks, (ii) wines and (iii) spirits. We calculated the daily consumption of alcohol in grams for each beverage by multiplying frequency with quantity, as defined in detail previously.^33^

### Statistical analyses

Descriptive statistics were conducted for background, PA and neighbourhood characteristics according to depressive symptoms. Due to the collinearity of the neighbourhood characteristics, ordinal logistic regression models were conducted separately for all neighbourhood variables using the four categories of depressive symptoms—none, mild, moderate and severe—as an outcome (model 1). Model 2 were adjusted for sex, marital status, harm avoidance, education, present smoking and alcohol intake. In model 3, self-reported or accelerometer-measured LPA was included along with other factors. In model 4, self-reported or accelerometer-measured MVPA was included. For PA, the odds ratios (ORs) were estimated per 60-min increases in LPA and MVPA; for population density, per inhabitant/0.1 ha increase; and for distance to the nearest grocery store, park or forest as well as for the length of cycle/pedestrian paths, the odds ratios were estimated per 1 km increase.

To account for attrition, we calculated weights based on sex, register-based marital status and register-based education (Supplementary table S1). Finally, we weighted all our ordinal logistic regression analyses using inverse probability weighting (*n* = 5408).

All the analyses were performed using IBM SPSS Statistics version 26. Figures were done using ArcGIS Pro 3.0.3 and R version 4.2.0, package tidyverse.

## Results

The characteristics of the total study population according to the severity of depressive symptoms are presented in table 1. Most of the participants were women (56.0%), married or in *de facto* relationship (78.8%) and had secondary education (68.5%). Those without depressive symptoms had a higher amount of self-reported and accelerometer-measured LPA and MVPA compared to those with any depressive symptoms. Characteristics of the participants with self-reported total LPA and with valid accelerometer-measured PA according to depressive symptoms are presented in Supplementary tables S2 and S3.

**Table 1:** Characteristics of the study population (*n* = 5489) according to the severity of depressive symptoms based on BDI-II

		Depressive symptoms			
Characteristics	Total	No depressive symptoms	Mild	Moderate	Severe
	(*n* = 5489)	(*n* = 4964)	(*n* = 321)	(*n* = 146)	(*n* = 58)
**Demographics**					
Sex					
Men	2416 (44.0)	2234 (45.0)	124 (38.6)	41 (28.1)	17 (29.3)
Women	3073 (56.0)	2730 (55.0)	197 (61.4)	105 (71.9)	41 (70.7)
Marital status					
Married/*de facto* relationship	4112 (78.8)	3773 (79.8)	216 (72.0)	93 (66.9)	30 (55.6)
Single/divorced/widowed	1109 (21.2)	953 (20.2)	84 (28.0)	46 (33.1)	24 (44.4)
Education					
Basic	125 (2.5)	104 (2.3)	8 (2.8)	8 (5.9)	5 (9.6)
Secondary	3475 (68.5)	3129 (68.1)	215 (74.1)	92 (67.6)	39 (75.0)
Tertiary	1474 (29.1)	1363 (29.7)	67 (23.1)	36 (26.5)	8 (15.4)
**Lifestyle factors and personality traits**					
Present smoking					
No	3698 (75.5)	3399 (76.7)	182 (64.1)	89 (67.4)	28 (52.8)
Yes	1202 (24.5)	1032 (23.3)	102 (35.9)	43 (32.6)	25 (47.2)
Alcohol intake (g/day)	11.4 (19.2)	10.8 (17.5)	15.8 (27.3)	18.4 (36.3)	17.3 (32.8)
Harm avoidance	13.2 (6.3)	12.5 (5.7)	19.1 (6.5)	21.8 (6.8)	24.4 (6.1)
**PA**					
Self-reported LPA (h/week)	2.7 (2.7)	2.7 (2.7)	2.3 (2.5)	2.4 (2.5)	2.1 (2.8)
Self-reported MVPA (h/week)	1.9 (2.0)	1.9 (2.0)	1.3 (1.8)	1.4 (1.8)	0.9 (1.1)
Accelerometer-measured LPA (h/week)	32.4 (8.4)	32.6 (8.4)	31.3 (8.5)	30.5 (10.1)	27.8 (7.5)
Accelerometer-measured MVPA (h/week)	8.0 (4.0)	8.1 (3.9)	7.5 (5.0)	7.2 (4.8)	6.5 (3.7)
Wear time h/day	16.3 (1.0)	16.3 (1.0)	16.3 (1.2)	16.0 (1.4)	16.0 (1.2)
**Neighbourhood characteristics**					
Population density (inhabitants/0.1 ha)	1.1 (1.5)	1.0 (1.4)	1.1 (1.6)	1.3 (1.8)	2.0 (3.0)
Distance to the closest grocery store (km)	3.1 (5.4)	3.1 (5.5)	2.9 (4.7)	3.5 (6.4)	1.6 (3.1)
Number of bus stops	4.3 (8.0)	4.2 (7.8)	4.1 (7.8)	6.1 (10.5)	7.6 (12.0)
Cycle/pedestrian paths (km)	11.4 (9.3)	11.3 (10.3)	11.6 (10.7)	13.7 (12.5)	15.6 (11.0)
Distance to the closest park (km)	19.4 (28.2)	19.6 (28.4)	18.5 (25.6)	18.4 (27.0)	11.0 (20.3)
Distance to the closest forest (km)	0.5 (0.6)	0.5 (0.6)	0.5 (0.6)	0.5 (0.6)	0.7 (0.7)
Level of urbanicity	−0.1 (2.5)	−0.2 (2.5)	0.01 (2.7)	0.6 (3.2)	1.7 (4.3)
Residential greenness	0.4 (0.1)	0.4 (0.1)	0.4 (0.2)	0.4 (0.2)	0.4 (0.2)

Notes: Values are mean (SD) or count (%). Values were calculated for the number of participants having data on the variable in question. BDI-II, Beck Depression Inventory-II; SD, standard deviation; PA, physical activity; LPA, light physical activity; MVPA, moderate-to-vigorous physical activity.

**Figure 2:**
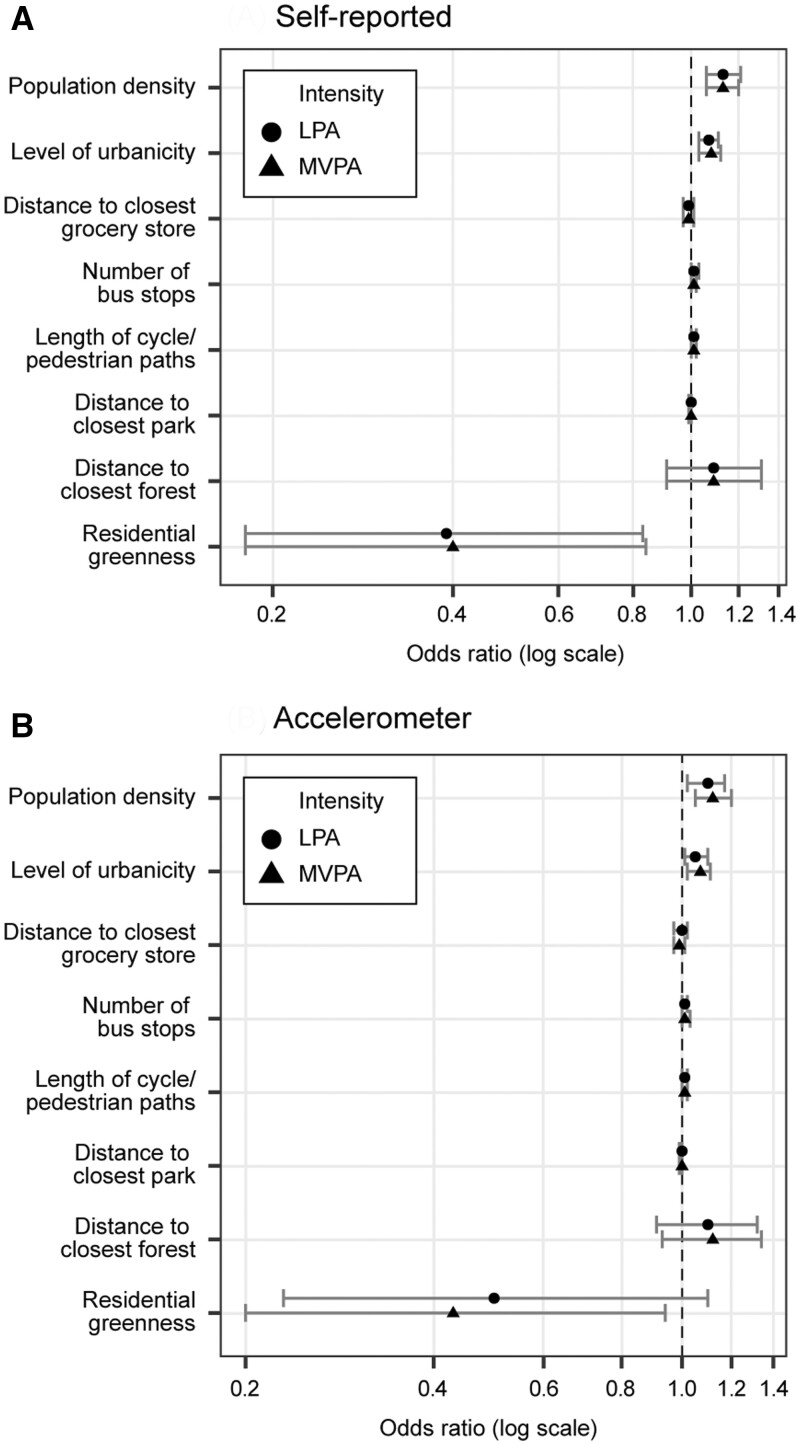
The associations between separate neighbourhood characteristics, (**A**) self-reported and (**B**) accelerometer-measured PA and depressive symptoms

The associations between separate neighbourhood characteristics, self-reported PA and severity of depressive symptoms according to ordinal logistic regression analyses are presented in figure 2A and Supplementary table S4 (models 1–4). In the final models (models 3 and 4), including LPA or MVPA as well as adjustment for all individual factors, higher population density [LPA model: OR = 1.13, 95% confidence interval (CI): 1.06, 1.21; MVPA model: OR = 1.13, 95% CI: 1.06, 1.20] and level of urbanicity (LPA model: OR = 1.07, 95% CI: 1.03, 1.11; MVPA model: OR = 1.08, 95% CI: 1.03, 1.12) were associated with a higher risk of experiencing more severe depressive symptoms. In addition, higher residential greenness (LPA model: OR = 0.39, 95% CI: 0.18, 0.83; MVPA model: OR = 0.40, 95% CI: 0.18, 0.84) was associated with a lower risk of experiencing more severe depressive symptoms. Higher self-reported MVPA was associated with a lower risk of experiencing more severe depressive symptoms in all the models.

The results of the ordinal logistic regression models for the associations between separate neighbourhood characteristics, accelerometer-measured PA and severity of depressive symptoms are presented in figure 2B and table 2. After including LPA and MVPA into the models (models 3 and 4), higher population density (LPA model: OR = 1.10, 95% CI: 1.02, 1.17; MVPA model: OR = 1.12, 95% CI: 1.05, 1.20) and level of urbanicity (LPA model: OR = 1.05 95% CI: 1.01, 1.10; MVPA model: OR = 1.07, 95% CI: 1.02, 1.11) were associated with a higher risk of experiencing more severe depressive symptoms. Higher residential greenness (OR = 0.43, 95% CI: 0.20, 0.94) was associated with a lower risk of experiencing more severe depressive symptoms in the MVPA model. Higher accelerometer-measured LPA was also associated with a lower risk of experiencing more severe depressive symptoms in all the models.

**Table 2:** Ordinal logistic regression analysis of separate neighbourhood characteristics and accelerometer-measured PA on severity of depressive symptoms (none, mild, moderate and severe) (*n *=* *5408)

	Model 1	Model 2	Model 3	Model 4
**Population density (residents/0.1 ha)**	1.10 (1.05–1.16)	1.12 (1.05–1.20)	1.10 (1.02–1.17)	1.12 (1.05–1.20)
Accelerometer-measured LPA			0.97 (0.96–0.99)	
Accelerometer-measured MVPA				0.98 (0.96–1.01)
**Distance to the closest grocery store (km)**	0.99 (0.98–1.01)	0.99 (0.97–1.01)	1.00 (0.97–1.02)	0.99 (0.97–1.01)
Accelerometer-measured LPA			0.97 (0.96–0.98)	
Accelerometer- measured MVPA				0.98 (0.96–1.01)
**Number of bus stops**	1.01 (1.00–1.02)	1.01 (1.00–1.03)	1.01 (1.00–1.02)	1.01 (1.00–1.03)
Accelerometer-measured LPA			0.97 (0.96–0.98)	
Accelerometer-measured MVPA				0.98 (0.95–1.01)
**Length of cycle/pedestrian paths (km)**	1.01 (1.00–1.02)	1.01 (1.00–1.02)	1.01 (1.00–1.02)	1.01 (1.00–1.02)
Accelerometer-measured LPA			0.97 (0.96–0.98)	
Accelerometer-measured MVPA				0.98 (0.96–1.01)
**Distance to the closest park (km)**	1.00 (0.99–1.00)	1.00 (0.99–1.00)	1.00 (0.99–1.00)	1.00 (0.99–1.00)
Accelerometer-measured LPA			0.97 (0.96–0.98)	
Accelerometer- measured MVPA				0.98 (0.96–1.01)
**Distance to the closest forest (km)**	1.16 (1.01–1.33)	1.09 (0.91–1.31)	1.10 (0.91–1.32)	1.12 (0.93–1.34)
Accelerometer-measured LPA			0.97 (0.96–0.98)	
Accelerometer-measured MVPA				0.98 (0.96–1.01)
**Residential greenness**	0.49 (0.28–0.87)	0.40 (0.19–0.85)	0.50 (0.23–1.10)	0.43 (0.20–0.94)
Accelerometer-measured LPA			0.97 (0.96–0.98)	
Accelerometer-measured MVPA				0.98 (0.96–1.01)
**Level of urbanicity**	1.08 (1.04–1.11)	1.07 (1.02–1.11)	1.05 (1.01–1.10)	1.07 (1.02–1.11)
Accelerometer-measured LPA			0.97 (0.96–0.99)	
Accelerometer-measured MVPA				0.98 (0.96–1.01)

Notes: Model 1: crude. Model 2: sex, marital status, harm avoidance, education, present smoking and alcohol intake. Model 3: sex, marital status, harm avoidance, education, present smoking, alcohol intake and accelerometer-measured LPA. Model 4: sex, marital status, harm avoidance, education, present smoking, alcohol intake and accelerometer-measured MVPA. Values are odds ratios and 95% confidence intervals. Values were calculated for the number of participants having data on the variable in question. PA, physical activity; LPA, light physical activity; MVPA, moderate-to-vigorous physical activity.

## Discussion

The main finding of this study was that participants living in residential areas with higher population density and urbanicity levels were at a higher risk of experiencing more severe depressive symptoms. Higher residential greenness was associated with a lower risk of experiencing more severe depressive symptoms irrespective of self-reported LPA or MVPA, accelerometer-measured MVPA and other individual factors. However, residential greenness was not significantly associated with depressive symptoms after adjustment for accelerometer-measured LPA. Higher accelerometer-measured LPA and higher self-reported MVPA were associated with lower risk of experiencing more severe depressive symptoms.

Our study partly supports the previously suggested finding that urban area characteristics are related to depressive symptoms,^7^^,^^8^ although in earlier studies the association has been inconsistent.^6^ Our result is in line with the study of Walters et al.^9^ suggesting an association between high population density and the prevalence of depression. These inconsistent findings could be partly attributed to the challenges encountered when studying the association between urbanicity and depression. Especially in cross-sectional settings, two major problems are observed, as discussed earlier by Sampson et al.^34^ First is the possibility of ‘healthy migrant effect’, which states that individuals who have migrated to urban areas might have better resources in the first place compared to those that stay in rural areas. Second, it is difficult to separate effects of living in urban area from other factors, such as initial reasons for migration. For example, people with depressive symptoms may have moved to urban areas where services are closer.^35^ Greenness has also been associated with lower depressive symptoms in earlier studies,^12^^,^^34^ in line with our findings, even though the distance to closest park or forest was not related to depressive symptoms in our models. Higher shares of green space can be found among Northern European Cities in contrast to Southern Europe,^36^ which may have affected our results. In the future, studies conducted in Northern Europe should differentiate between urban areas with high and low amounts of green space and also consider the qualitative features of green space.

In this study, we found that higher accelerometer-measured LPA and higher self-reported MVPA were associated with lower risk of experiencing more severe depressive symptoms. Though accelerometers are relatively more reliable PA measurement methods, their ability for measuring certain activity types such as standing and light-intensity PA remain limited. On the other hand, self-reports may not accurately capture all levels of PA, but rather give insight on the type and self-perceived strenuousness of activity.^37^ Due to recall and social desirability bias, PA questionnaires tend to overestimate, especially higher intensity PA.^38^ In this study, self-reports covered only leisure-time activities, whereas accelerometer also measures work and commuting, for example. There is a possibility that those participating in accelerometer data collection were more active than those who only filled in the PA questionnaire. Considering the differences in the measurement methods, it is important to include both measurements of PA when possible, as recommended previously.^20^

Our study has several strengths. This was the first population-based study to evaluate the relationships between objectively measured residential environments, using both self-reported and device-based measures of PA and depressive symptoms. In addition, we used a wide set of neighbourhood characteristics created using Geographic Information System as well as relatively large, representative, population-based cohort data. In addition, compliance with activity monitor wear was high. All these data allowed us to explore whether the associations between environmental characteristics and depressive symptoms differed after including PA measured with two complementary approaches. To ensure that the analysis accurately represents the population, we also used weights based on sex, marital status and education in our analysis.

Nevertheless, this study has some limitations. First, this was a cross-sectional study; therefore, we were unable to conclude the causal relationship between environment, PA and depressive symptoms. In addition, although we adjusted for a relatively comprehensive set of confounders, the potential confounding effects of other unmeasured factors, such as chronic diseases, remained. Second, even though we weighted the analysis, some biases due to attrition remained. Third, given that socio-economic wellbeing is divided regionally, it may have affected our results. Fourth, we used self-assessed depressive symptoms instead of more robust indicators, such as hospitalization. Fifth, accelerometers were used to monitor total PA behaviour without further differentiation between leisure-time and work-related PA. From the questionnaire, only items concerning leisure-time LPA and MVPA were included due to the lack of suitable questionnaire items regarding occupational PA. Leisure-time and occupational PA may be associated differently with depressive symptoms, which should be considered in future studies. Sixth, when considering the neighbourhood characteristics, we were unable to assess the quality of the environment, which could be a considerable factor when it comes to green areas^39^ or urban environments.^40^ Last is the chosen buffer size, which should be considered when using neighbourhood characteristics, and this may have affected our results. When comparing our results to other studies, it should be noted that definitions of urban and rural areas usually vary in different countries.

In conclusion, this population-based birth cohort study provides a comprehensive view of the association between living environment, PA and depressive symptoms. In almost all models, higher population density and level of urbanicity were associated with higher risk of experiencing more severe depressive symptoms and higher residential greenness with lower risk of having more severe symptoms, even after adjustments for self-reported LPA and MVPA and accelerometer-measured LPA and MVPA. Higher accelerometer-measured LPA and self-reported MVPA were associated with lower risk of having more severe depressive symptoms. It appears that both residential environment and PA behaviour play a role in depressive symptoms; however, further research among populations of different ages are required.

## Supplementary Material

ckad215_Supplementary_Data

## Data Availability

NFBC data are available from the University of Oulu, Infrastructure for Population Studies. Permission to use the data can be applied for research purposes via an electronic material request portal. In the use of data, we followed the EU general data protection regulation (679/2016) and the Finnish Data Protection Act. The use of personal data is based on a cohort participant’s written informed consent in their latest follow-up study, which may cause limitations to its use. Please contact the NFBC project centre (NFBCprojectcenter@oulu.fi) and visit the cohort website (www.oulu.fi/nfbc) for more information. Key points Higher population density and urbanicity level were associated with a higher risk of experiencing more severe depressive symptoms irrespective of physical activity (PA) level. Higher residential greenness was associated with a lower risk of experiencing more severe depressive symptoms irrespective of PA level. Higher self-reported moderate-to-vigorous PA and accelerometer-measured leisure-time light PA were independently associated with a lower risk of experiencing more severe depressive symptoms. Findings of the present study can be utilized when designing interventions for the prevention of depression.
