# Supplementary material for: Associations between neighbourhood characteristics, physical activity and depressive symptoms: the Northern Finland Birth Cohort 1966 Study
Source: Eur J Public Health. 2023 Dec 11;34(1):114–20. doi: 10.1093/eurpub/ckad215 (PMC10843961; doi:10.1093/eurpub/ckad215)
Supplement: ckad215_Supplementary_Data [file ckad215_supplementary_data.docx]

**Associations between Neighbourhood Characteristics, Physical Activity and Depressive Symptoms: The Northern Finland Birth Cohort 1966 Study**

Nina Rautio†, Marjo Seppänen†, Markku Timonen, Soile Puhakka, Mikko Kärmeniemi, Jouko Miettunen, Tiina Lankila, Vahid Farrahi, Maisa Niemelä, Raija Korpelainen

^†^These authors contributed equally to this work.

**SUPPLEMENTARY TABLES**

**Table S1.** Counts (%) and corresponding weights for participants of the 46-year-old study based on sex, register-based education and register-based marital status categories.

| Education | Sex | Marital status | Full sample | | Weight |
| --- | --- | --- | --- | --- | --- |
|  |  |  | **n** | **%** |  |
| Basic | Men | Married | 72 | 32.4 | 1.651 |
| Basic | Men | Not married | 97 | 25.1 | 2.131 |
| Basic | Women | Married | 49 | 40.5 | 1.321 |
| Basic | Women | Not married | 58 | 27.6 | 1.938 |
| Secondary | Men | Married | 674 | 51.1 | 1.047 |
| Secondary | Men | Not married | 507 | 37.4 | 1.430 |
| Secondary | Women | Married | 672 | 59.8 | 0.895 |
| Secondary | Women | Not married | 518 | 52.5 | 1.019 |
| Tertiary | Men | Married | 717 | 59.7 | 0.896 |
| Tertiary | Men | Not married | 315 | 50.9 | 1.051 |
| Tertiary | Women | Married | 1122 | 69.8 | 0.766 |
| Tertiary | Women | Not married | 607 | 64 | 0.836 |
|  |  | **Total** | 5408 | 53.5 | 1.000 |

**Table S2.** Characteristics of the participants with self-reported leisure time total PA (n=4936) according to the severity of depressive symptoms based on BDI-II.

|  | **Depressive symptoms** | | | |
| --- | --- | --- | --- | --- |
| **Characteristics** | **No depressive symptoms**  **(n=4463)** | **Mild**  **(n=286)** | **Moderate (n=134)** | **Severe**  **(n=53)** |
| **Demographics** |  |  |  |  |
| *Sex* |  |  |  |  |
| Men | 1957 (43.8) | 109 (38.1) | 36 (26.9) | 15 (28.3) |
| Women | 2506 (56.2) | 177(61.9) | 98 (73.1) | 38 (71.7) |
| *Marital status* |  |  |  |  |
| Married/de facto relationship | 3562 (80.1) | 205 (72.2) | 89 (66.4) | 29 (54.7) |
| Single/divorced/widowed | 887 (19.9) | 79 (27.8) | 45 (33.6) | 24 (45.3) |
| *Education* |  |  |  |  |
| Basic | 96 (2.2) | 7 (2.6) | 8 (6.1) | 5 (9.8) |
| Secondary | 2940 (68.0) | 206 (75.2) | 89 (67.4) | 38 (74.5) |
| Tertiary | 1285 (29.7) | 61 (22.3) | 35 (26.5) | 8 (15.7) |
| **Lifestyle factors and personality traits** |  |  |  |  |
| *Present smoking* |  |  |  |  |
| No | 3206 (76.8) | 170 (62.7) | 85 (66.4) | 27 (51.9) |
| Yes | 967 (23.2) | 101 (37.3) | 43 (33.6) | 25 (48.1) |
| *Alcohol intake (g/day)* | 10.8 (17.6) | 15.8 (27.9) | 18.0 (36.5) | 17.5 (33.1) |
| *Harm avoidance* | 12.5 (5.8) | 19.2 (6.5) | 22.0 (6.9) | 24.3 (6.2) |
| **PA** |  |  |  |  |
| *Self-reported LPA (h/week)* | 2.7 (2.7) | 2.3 (2.5) | 2.4 (2.5) | 2.1 (2.8) |
| *Self-reported MVPA (h/week)* | 1.9 (2.0) | 1.2 (1.7) | 1.3 (1.8) | 0.9 (1.1) |
| *Accelerometer-measured LPA (h/week)* | 32.6 (8.4) | 31.2 (8.6) | 30.7 (10.3) | 28.1 (7.2) |
| *Accelerometer-measured MVPA (h/week)* | 8.0 (3.9) | 7.6 (5.2) | 7.1 (4.9) | 6.4 (3.7) |
| *Wear time h/day* | 16.3 (0.9) | 16.3 (1.2) | 15.9 (1.4) | 16.1 (1.0) |
| **Neighbourhood characteristics** |  |  |  |  |
| *Population density (inhabitants/0.1ha)* | 1.0 (1.4) | 1.2 (1.6) | 1.3 (1.8) | 1.9 (3.1) |
| *Distance to the closest grocery store (km)* | 3.1 (5.5) | 2.9 (4.6) | 3.7 (6.6) | 1.7 (3.2) |
| *Number of bus stops* | 4.2 (7.8) | 4.3 (8.1) | 5.7 (10.1) | 6.4 (11.4) |
| *Cycle/pedestrian paths (km)* | 11.3 (10.3) | 11.8 (11.0) | 13.3 (12.5) | 14.5 (10.5) |
| *Distance to the closest park (km)* | 19.5 (28.1) | 18.4 (25.4) | 18.8 (26.8) | 12.0 (20.9) |
| *Distance to the closest forest (km)* | 0.5 (0.6) | 0.5 (0.6) | 0.5 (0.6) | 0.7 (0.6) |
| *Level of urbanicity* | -0.2 (2.4) | 0.1 (2.8) | 0.5 (3.2) | 1.6 (4.4) |
| *Residential greenness* | 0.4 (0.1) | 0.4 (0.2) | 0.4 (0.2) | 0.4 (0.2) |

Values are mean (SD) or count (%). Values were calculated for the number of participants having data on the variable in question.

PA: physical activity; BDI-II: Beck Depression Inventory-II; SD: standard deviation; LPA: light physical activity; MVPA: moderate-to-vigorous physical activity.

**Table S3.** Characteristics of the participants with valid accelerometer data (n=5193) according to the severity of depressive symptoms based on BDI-II.

|  | **Depressive symptoms** | | | |
| --- | --- | --- | --- | --- |
| **Characteristics** | **No depressive symptoms**  **(n=4709)** | **Mild**  **(n=299)** | **Moderate**  **(n=131)** | **Severe**  **(n=54)** |
| **Demographics** |  |  |  |  |
| *Sex* |  |  |  |  |
| Men | 2119 (45.0) | 118 (39.5) | 37 (28.2) | 14 (25.9) |
| Women | 2590 (55.0) | 181 (60.5) | 94 (71.8) | 40 (74.1) |
| *Marital status* |  |  |  |  |
| Married/de facto relationship | 3612 (80.1) | 205 (73.0) | 88 (68.5) | 30 (57.7) |
| Single/divorced/widowed | 898 (19.9) | 76 (27.0) | 40 (31.5) | 22 (42.3) |
| *Education* |  |  |  |  |
| Basic | 96 (2.2) | 8 (2.9) | 7 (5.6) | 4 (8.0) |
| Secondary | 2989 (68.1) | 202 (74.3) | 83 (66.9) | 38 (76.0) |
| Tertiary | 1303 (29.7) | 62 (22.8) | 34 (27.4) | 8 (16.0) |
| **Lifestyle factors and personality traits** |  |  |  |  |
| *Present smoking* |  |  |  |  |
| No | 3256 (77.1) | 170 (63.9) | 84 (70.0) | 27 (52.9) |
| Yes | 967 (22.9) | 96 (36.1) | 36 (30.0) | 24 (47.1) |
| *Alcohol intake (g/day)* | 10.6 (17.0) | 15.6 (26.5) | 18.7 (37.3) | 16.9 (32.9) |
| *Harm avoidance* | 12.5 (5.7) | 18.9 (6.5) | 21.5(6.8) | 24.3 (6.2) |
| **PA** |  |  |  |  |
| *Self-reported LPA (h/week)* | 2.7 (2.7) | 2.3 (2.5) | 2.5 (2.6) | 2.1 (2.8) |
| *Self-reported MVPA (h/week)* | 1.9 (2.0) | 1.3 (1.7) | 1.4 (1.8) | 0.9 (1.1) |
| *Accelerometer-measured LPA (h/week)* | 32.6 (8.4) | 31.3 (8.6) | 30.5 (10.1) | 27.8 (7.5) |
| *Accelerometer-measured MVPA (h/week)* | 8.0 (3.9) | 7.5 (5.0) | 7.2 (4.9) | 6.5 (3.7) |
| *Wear time h/day* | 16.3 (1.0) | 16.3 (1.2) | 16.0 (1.4) | 16.0 (1.0) |
| **Neighbourhood characteristics** |  |  |  |  |
| *Population density (inhabitants/0.1ha)* | 1.0 (1.4) | 1.1 (1.6) | 1.3 (1.8) | 2.0 (3.1) |
| *Distance to the closest grocery store (km)* | 3.1 (5.5) | 2.9 (4.6) | 3.7 (6.7) | 1.6 (3.2) |
| *Number of bus stops* | 4.2 (7.8) | 3.9 (7.6) | 6.0 (10.6) | 7.3 (11.6) |
| *Cycle/pedestrian paths (km)* | 11.2 (10.2) | 11.3 (10.6) | 13.6 (12.7) | 15.4 (11.1) |
| *Distance to the closest park (km)* | 19.8 (28.7) | 18.5 (25.5) | 19.5 (28.0) | 11.8 (20.8) |
| *Distance to the closest forest (km)* | 0.5 (0.6) | 0.5 (0.6) | 0.5 (0.6) | 0.7 (0.7) |
| *Level of urbanicity* | -0.2 (2.4) | -0.0 (2.7) | 0.5 (3.2) | 1.7 (4.4) |
| *Residential greenness* | 0.4 (0.1) | 0.4 (0.2) | 0.4 (0.2) | 0.4 (0.2) |

Values are mean (SD) or count (%). Values were calculated for the number of participants having data on the variable in question.

BDI-II: Beck Depression Inventory-II; SD: standard deviation; PA: physical activity; LPA: light physical activity; MVPA: moderate-to-vigorous physical activity.

**Table S4.** Ordinal logistic regression analysis of separate neighbourhood characteristics and self-reported measured PA on the severity of depressive symptoms (none, mild, moderate and severe) (*n*=5489).

|  | **Model 1** | **Model 2** | **Model 3** | **Model 4** |
| --- | --- | --- | --- | --- |
| **Population density**  **(residents/0.1 ha)** | 1.10 (1.05–1.16) | 1.12 (1.05–1.20) | 1.13 (1.06–1.21) | 1.13 (1.06–1.20) |
| Self-reported LPA |  |  | 0.95 (0.91–1.00) |  |
| Self-reported MVPA |  |  |  | 0.89 (0.83–0.96) |
| **Distance to the closest**  **grocery store (km)** | 0.99 (0.98–1.01) | 0.99 (0.97–1.01) | 0.99 (0.97–1.01) | 0.99 (0.97–1.01) |
| Self-reported LPA |  |  | 0.95 (0.91–1.00) |  |
| Self-reported MVPA |  |  |  | 0.89 (0.83–0.96) |
| **Number of bus stops** | 1.01 (1.00–1.02) | 1.01 (1.00–1.03) | 1.01 (1.00–1.03) | 1.01 (1.00–1.02) |
| Self-reported LPA |  |  | 0.96 (0.91–1.00) |  |
| Self-reported MVPA |  |  |  | 0.89 (0.83–0.96) |
| **Length of cycle/**  **pedestrian paths (km)** | 1.01 (1.00–1.02) | 1.01 (1.00–1.02) | 1.01 (1.00–1.02) | 1.01 (1.00–1.02) |
| Self-reported LPA |  |  | 0.95 (0.91–1.00) |  |
| Self-reported MVPA |  |  |  | 0.89 (0.83–0.96) |
| **Distance to the closest**  **park (km)** | 1.00 (0.99–1.00) | 1.00 (0.99–1.00) | 1.00 (0.99–1.00) | 1.00 (0.99–1.00) |
| Self-reported LPA |  |  | 0.95 (0.91–1.00) |  |
| Self-reported MVPA |  |  |  | 0.89 (0.83–0.96) |
| **Distance to the closest**  **forest (km)** | 1.16 (1.01–1.33) | 1.09 (0.91–1.31) | 1.09 (0.91–1.31) | 1.09 (0.91–1.31) |
| Self-reported LPA |  |  | 0.95 (0.91–1.00) |  |
| Self-reported MVPA |  |  |  | 0.89 (0.83–0.96) |
| **Residential greenness** | 0.49 (0.28–0.87) | 0.40 (0.19–0.85) | 0.39 (0.18–0.83) | 0.40 (0.18–0.84) |
| Self-reported LPA |  |  | 0.95 (0.91–1.00) |  |
| Self-reported MVPA |  |  |  | 0.89 (0.83–0.96) |
| **Level of urbanicity** | 1.08 (1.04–1.11) | 1.07 (1.02–1.11) | 1.07 (1.03–1.11) | 1.08 (1.03–1.12) |
| Self-reported LPA |  |  | 0.95 (0.91–1.00) |  |
| Self-reported MVPA |  |  |  | 0.89 (0.83–0.95) |

Model 1: crude

Model 2: sex, marital status, harm avoidance, education, present smoking and alcohol intake.

Model 3: sex, marital status, harm avoidance, education, present smoking, alcohol intake and self-reported LPA.

Model 4: sex, marital status, harm avoidance, education, present smoking, alcohol intake and self-reported MVPA.

Values are odds ratios and 95% confidence intervals. Values were calculated for the number of participants having data on the variable in question.

PA: physical activity; LPA: light physical activity; MVPA: moderate-to-vigorous physical activity.
